# Decreased B1 and B2 Lymphocytes Are Associated With Mortality in Elderly Patients With Chronic Kidney Diseases

**DOI:** 10.3389/fmed.2020.00075

**Published:** 2020-03-20

**Authors:** Jieshan Lin, Wenfang Tang, Wei Liu, Feng Yu, Yanhua Wu, Xiaowu Fang, Maohua Zhou, Wenke Hao, Wenxue Hu

**Affiliations:** ^1^Department of Nephrology, Guangdong Provincial People's Hospital, Guangdong Academy of Medical Sciences, Guangdong Provincial Geriatrics Institute, Guangzhou, China; ^2^Shantou University Medical College, Shantou, China; ^3^Department of Laboratory Medicine, Guangdong Provincial People's Hospital, Guangdong Academy of Medical Sciences, Guangzhou, China

**Keywords:** B cells, elderly, chronic kidney disease, correlation, prognosis

## Abstract

**Aim:** Loss of renal function is associated with immune deficiency; however, few studies have addressed the role of B lymphocytes in elderly patients with chronic kidney disease (CKD). In this study, we examined the distribution and the relationship of the B lymphocyte subpopulation with clinical outcomes in elderly CKD patients.

**Methods:** In this study, a total of 380 patients (312 CKD patients and 68 non-CKD controls) were recruited. Venous blood samples were analyzed by flow cytometry to determine the following B cell subsets: total B cells (CD19+), innate B1 cells (CD19+CD5+), and conventional B2 cells (CD19+CD5–). Correlations between the B cell subsets with clinical features and patient prognosis were analyzed.

**Results:** A total of 380 patients (mean age 82.29 ± 6.22 years, 76.3% male) were included. The median follow-up time was 37.0 months (range, 1–109 months); 109 (28.7%) patients died. The main causes of death were infections (59.6%) and cardiovascular diseases (22.9%). Correlation analysis showed that levels of serum creatinine (SCr), blood urea nitrogen (BUN), and CKD were negatively associated with B1 cells. However, lymphocytes, T lymphocytes, and estimated glomerular filtration rate (eGFR) were positively correlated with B1 cells (all *P* < 0.05). B2 cells were negatively associated with age, SCr, cystatin C, BUN, and CKD, and were positively correlated with hemoglobin, lymphocytes, T lymphocytes, NK cells, and eGFR (all *P* < 0.05). Patient survival was significantly better in patients with B cells > 0.05 × 10^9^/L, B1 cells > 0.02 × 10^9^/L, and B2 cells > 0.04 × 10^9^/L. Multivariate Cox regression analysis showed that B1 cells > 0.02 × 10^9^/L [hazard ratio (HR) = 0.502, 95% confidence interval (CI): 0.297–0.851, *P* = 0.010] and B2 cells > 0.04 × 10^9^/L (HR = 0.536, 95% CI: 0.319–0.901, *P* = 0.019) were independent protective factors for all-cause mortality.

**Conclusions:** Our results showed that B1 and B2 cells exhibited a significantly negative correlation with the progression of CKD in elderly patients. Moreover, B1 and B2 cells were independent prognostic factors for survival, which indicates that the decrease in B cells may be associated with the progression of kidney diseases.

## Introduction

Reduced renal function is associated with cardiovascular events, infections and death in chronic kidney disease (CKD) patients, and the risk increases when CKD progresses to end-stage renal disease (ESRD) (1). It is believed to be related to abnormalities of the immune system, mainly systemic inflammation, and immune deficiency. Systemic inflammation contributes to atherosclerosis, cardiovascular disease, cachexia, and anemia. Immune deficiencies include decreased granulocyte and monocyte/macrophage phagocytic function, defective antigen presentation by monocytes/macrophages, reduced antibody production by B lymphocytes and impaired T-cell-mediated immunity, and they can lead to a low response to vaccination, an increased incidence of microbial infections, tumors, and delayed hypersensitivity (2, 3).

B lymphocytes (CD19+) regulate several immune responses and inflammatory processes. They arise from hematopoietic stem cells and express a diverse repertoire of immunoglobulins against a wide array of pathogens and function as antigen-presenting cells to T lymphocytes (4). There are two groups of B cells, innate B1 cells (CD19+CD5+) and conventional B2 (CD19+CD5–) cells. B1 cells predominate in peritoneal and pleural cavities, and they are considered to be elements of the innate immune system and account for 25–27% of peripheral blood B lymphocytes (5). They produce mainly IgM antibodies that have high cross-reactivity but low affinity. These antibodies constitute a readily available pool of immunoglobulins for use against a variety of infections before specific high-affinity antibodies are produced. In contrast, B2 cells account for 75–80% of peripheral blood B lymphocytes. B2 cells must differentiate into plasma cells, which are responsible for developing an adaptive response, and they produce diverse and high-affinity antibodies (6).

Several studies have indicated a reduction in peripheral blood B lymphocytes in patients with ESRD (7, 8). The dysfunctional immune system has a substantial clinical impact on both the morbidity and mortality of ESRD patients (9, 10). However, the results remain controversial, and most of these studies were restricted to ESRD patients or did not pay attention to elderly patients. The aim of this study was to investigate the correlation between peripheral blood B lymphocyte populations and mortality in elderly CKD patients.

## Materials and Methods

### Patients

Inclusion criteria: Patients aged ≥ 60 years old with a diagnosis of CKD met the criteria for inclusion. The control group (68 patients) without CKD included patients hospitalized for hypertension, prostatic hyperplasia, and osteoporosis. All patients had steady-state kidney function for more than 3 months. CKD is defined as abnormalities of kidney structure or function, present for > 3 months, with implications for health. The criteria for CKD are as follows: (1) markers of kidney damage (>1 for >3 months): albuminuria, urinary sediment abnormalities, electrolyte, and other abnormalities due to tubular disorders, abnormalities detected by histology, structural abnormalities detected by imaging, history of kidney transplantation, and/ or (2) decreased GFR (for >3 months): GFR <60 ml/min/1.73 m^2^. CKD was classified by estimated glomerular filtration rate (eGFR) from stage 1 to stage 5: stage 1 or stage 2 was defined as an eGFR ≥ 90 or 60–89 ml/min/1.73 m^2^ with kidney damage; stage 3 was defined as an eGFR from 30 to 59 ml/min/1.73 m^2^; stage 4 was defined as an eGFR from 15 to 29 ml/min/1.73 m^2^; and stage 5 was defined as an eGFR under 15 ml/min/1.73 m^2^ (11).

Exclusion criteria: (1) patients with acute kidney injury; (2) patients with evidence of acute infection, active malignancy, autoimmune disease, hematological disorder, and thyroid malfunction; (3) patients who had started renal replacement therapy (RRT); (4) patients who received corticosteroid therapy; and (5) patients who lacked laboratory or clinical data.

A total of 312 CKD patients aged ≥ 60 years were retrospectively studied in Guangdong Provincial People's Hospital from January 2010 to December 2018. For all patients, we recorded clinical data, including sex and age, hemoglobin, cystatin C, uric acid, serum creatinine (SCr), blood urea nitrogen (BUN), serum albumin (ALB), protein/creatinine ratio, proteinuria, cholesterol, triglyceride, white blood cell (WBC), neutrophil, monocytes, and lymphocytes levels; and comorbidities such as hypertension and diabetes. SCr was measured by the enzymatic method, and cystatin C was determined by particle-enhanced turbidimetric immunoassay (PETIA). The study involving human participants was approved by the Ethical Committee of Guangdong Provincial People's Hospital.

### Definitions and Study Endpoints

eGFR (ml/min/1.73 m^2^) was calculated using the Chronic Kidney Disease Epidemiology collaboration equation (CKD-EPI) (12). CKD was diagnosed and categorized according to the Kidney Disease Improving Global Outcomes (KDIGO) guidelines. The median follow-up duration of the study was 37.0 months. The primary endpoint of this study was all-cause mortality.

### Flow Cytometry Analysis (FCM)

**Figure 1:**
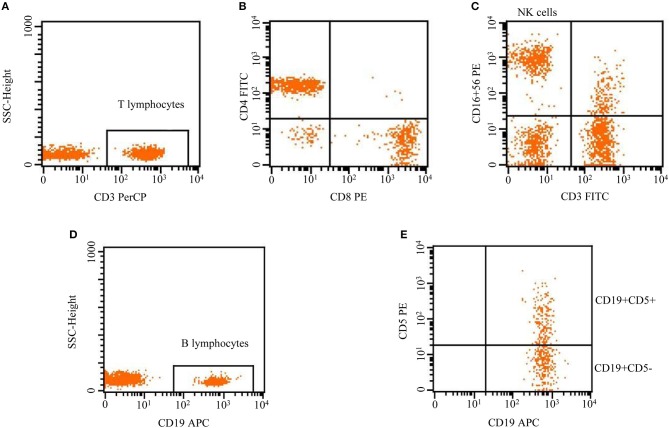
Flow cytometric analysis of lymphocytes, including T lymphocytes (CD3+), NK cells (CD3–CD16+CD56+), and B lymphocytes (CD19+). B cells were divided into B1 and B2 cells according to the surface expression of CD5+, CD19+CD5+ (innate B1 cells), and CD19+CD5– (conventional B2 cells).

T lymphocytes (CD3+), NK cells (CD3–CD16+CD56+), B lymphocytes (CD19+), B1 lymphocytes (CD19+CD5+), and B2 lymphocytes (CD19+CD5–) were analyzed by flow cytometry. Peripheral blood samples obtained by venipuncture were collected in EDTA anticoagulant. A total of 100 μl of whole blood was incubated with 10 μl of anti-human CD3-PerCP, CD4-FITC, CD8-PE, CD16+CD56PE, CD19-APC, and CD5-PE antibodies for 20 min at 4°C in the dark. A total of 2 ml of red blood cell lysis buffer was added before staining to each tube, vortexed well, and incubated for 10 min at room temperature in the dark. Then, the cells were washed twice with phosphate buffer saline (PBS), and the supernatant was discarded. The cell pellet was dissolved in 300 μl of 1% paraformaldehyde (PFD). Last, 20,000 cells were acquired by using FACS (BD USA) and were analyzed using Cellquest-Pro analysis software to determine the subpopulation counts (Figure 1).

### Statistical Analysis

Statistical analyses were performed using SPSS (version 20.0; SPSS Inc., Chicago, IL, USA) and GraphPad Prism (version 5.0; GraphPad Software, Inc., La Jolla, CA, USA). Fisher's exact test or the χ^2^ test was used to compare qualitative data, and the Wilcoxon rank-sum test was used for continuous variables. Pearson's or Spearman's test were used for the correlation between different B cells and clinical data. A *P* value < 0.05 was regarded as significant.

We utilized X-tile software version 3.6.1 (Yale University, New Haven, USA) to determine the expression of CD19+ B cells, CD19+CD5+ B1 cells, and CD19+CD5– B2 cells and the optimization of cutoff points based on outcome; and the cutoff points from X-tile were 0.05 × 10^9^/L, 0.02 × 10^9^/L, and 0.04 × 10^9^/L, respectively (13). The Kaplan–Meier method with the log-rank test was utilized for survival analysis, and Cox regression analysis was used for univariate and multivariate analyses.

## Results

### Patient and Demographic Details

There were 380 patients entered in the final analysis: 68 control subjects, 75 patients with CKD1-2, 131 patients with CKD3, 49 patients with CKD4, and 57 patients with CKD5. The majority of patients were male (76.3%), and the mean age was 82.29 years, ranging from 60 to 97 years. Furthermore, 309 (81.3%) patients had hypertension, and 145 (38.2%) had diabetes. The mean eGFR was 49.62 ± 26.69 ml/min/1.73 m^2^, and the average levels of CD19+ B cells, CD19+CD5+ B1 cells, and CD19+CD5– B2 cells were (0.16 ± 0.20) × 10^9^/L, (0.06 ± 0.16) × 10^9^/L, and (0.10 ± 0.08) × 10^9^/L, respectively. In addition, the average level of T lymphocytes and NK cells was (1.02 ± 0.43) × 10^9^/L and (0.32 ± 0.20) × 10^9^/L. The median follow-up time was 37.0 months (range, 1–109 months). There were 109 (28.7%) deaths at the end of follow-up, of which 65 (59.6%) were attributed to infections and 25 (22.9%) to cardiovascular diseases. Other causes of death included tumors (5 patients, 4.6%), cerebral hemorrhage (5 patients, 4.6%), sudden death (5 patients, 4.6%), and others (4 patients, 3.7%). The patient characteristics are described in Table 1.

**Table 1:** Patient characteristics.

**Variable**	***n* (%) or mean ± SD**
Age (years)	82.29 ± 6.22
Sex (M/F)	290/90
Group control	68 (17.9%)
CKD1-2	75 (19.7%)
CKD3	131 (34.5%)
CKD4	49 (12.9%)
CKD5	57 (15.0%)
Hypertation, *n* (%)	309 (81.3%)
Diabetes, *n* (%)	145 (38.2%)
Hemoglobin (g/L)	116.82 ± 20.30
SCr (μmol/L)	174.08 ± 159.90
eGFR _CKD−EPI_ (ml/min/1.73 m^2^)	49.62 ± 26.69
BUN (mmol/L)	9.83 ± 7.73
Cystre-C (mg/L)	2.28 ± 2.35
Uric acid (μmol/L)	395.34 ± 115.34
Albumin (g/L)	34.16 ± 7.92
protein/creatinine ratio (mg/g Cr)	1203.29 ± 2447.34
Proteinuria (mg/day)	762.71 ± 1326.84
Cholesterol (mmol/L)	4.38 ± 1.19
Triglyceride (mmol/L)	1.32 ± 1.20
WBC (10^9^/L)	6.49 ± 2.03
Neutrophil (10^9^/L)	4.18 ± 1.69
Monocytes (10^9^/L)	0.57 ± 0.27
Lymphocytes (10^9^/L)	1.56 ± 0.66
T lymphocytes (10^9^/L)	1.02 ± 0.43
NK cells (10^9^/L)	0.32 ± 0.20
B lymphocytes (10^9^/L)	0.16 ± 0.20
B1 lymphocytes (10^9^/L)	0.06 ± 0.16
B2 lymphocytes (10^9^/L)	0.10 ± 0.08

*CKD, chronic kidney disease; eGFR, estimated glomerular filtration rate; SCr, serum creatinine; BUN, blood urea nitrogen; WBC, white blood cell*.

### The Correlation Between Different B Cells and Clinical Data

We analyzed CD19+ B cells, CD19+CD5+ B1 cells, and CD19+CD5– B2 cells with the clinical data and found that SCr (*r* = −0.110, *P* = 0.032), BUN (*r* = −0.113, *P* = 0.028), and CKD (*r* = −0.318, *P* < 0.001) were negatively correlated with B1 cells. However, lymphocytes (*r* = 0.449, *P* < 0.001), T lymphocytes (*r* = 0.211, *P* < 0.001), and the eGFR (*r* = 0.140, *P* = 0.006) were positively correlated with B1 cells. B2 cells were negatively associated with age (*r* = −0.150, *P* = 0.003), SCr (*r* = −0.153, *P* = 0.003), cystatin C (*r* = −0.127, *P* = 0.022), BUN (*r* = −0.158, *P* = 0.002), and CKD (*r* = −0.187, *P* < 0.001), and were positively correlated with hemoglobin (*r* = 0.105, *P* = 0.042), lymphocytes (*r* = 0.596, *P* < 0.001), T lymphocytes (*r* = 0.474, *P* < 0.001), NK cells (*r* = 0.200, *P* < 0.001), and the eGFR (*r* = 0.142, *P* = 0.006). Total B cells (CD19+) were negatively correlated with SCr (*r* = −0.149, *P* = 0.004), cystatin C (*r* = −0.119, *P* = 0.032), BUN (*r* = −0.153, *P* = 0.003), and CKD (*r* = −0.241, *P* < 0.001), and were positively correlated with lymphocytes (*r* = 0.593, *P* < 0.001), T lymphocytes (*r* = 0.352, *P* < 0.001), NK cells (*r* = 0.144, *P* = 0.005), and the eGFR (*r* = 0.169, *P* = 0.001) (Table 2).

**Table 2:** Correlation between different B cells with the clinical data.

**Variables**	**B cells (10^**9**^/L)**	**B1 cells (10^**9**^/L)**	**B2 cells (10^**9**^/L)**
Age	*r* = −0.094	*r* = −0.047	*r* = −0.150
	*P* = 0.069	*P* = 0.364	*P* = 0.003^*^
Systolic pressure	*r* = −0.007	*r* = 0.02	*r* = −0.059
	*P* = 0.893	*P* = 0.704	*P* = 0.251
Diastolic pressure	*r* = −0.030	*r* = −0.014	*r* = −0.048
	*P* = 0.556	*P* = 0.783	*P* = 0.353
Hemoglobin (g/L)	*r* = 0.100	*r* = 0.072	*r* = 0.105
	*P* = 0.052	*P* = 0.162	*P* = 0.042^*^
Lymphocytes (10^9^/L)	*r* = 0.593	*r* = 0.449	*r* = 0.596
	*P* < 0.001^*^	*P* < 0.001^*^	*P* < 0.001^*^
Neutrophil (10^9^/L)	*r* = −0.067	*r* = −0.053	*r* = −0.062
	*P* = 0.194	*P* = 0.303	*P* = 0.234
Monocytes (10^9^/L)	*r* = 0.011	*r* = −0.019	*r* = 0.069
	*P* = 0.838	*P* = 0.717	*P* = 0.178
T lymphocytes (10^9^/L)	*r* = 0.352	*r* = 0.211	*r* = 0.474
	*P* < 0.001^*^	*P* < 0.001^*^	*P* < 0.001^*^
NK cells (10^9^/L)	*r* = 0.144	*r* = 0.084	*r* = 0.200
	*P* = 0.005^*^	*P* = 0.105	*P* < 0.001^*^
SCr (μmol/L)	*r* = −0.149	*r* = −0.110	*r* = −0.153
	*P* = 0.004^*^	*P* = 0.032^*^	*P* = 0.003^*^
Cystre-C (mg/L)	*r* = −0.119	*r* = −0.086	*r* = −0.127
	*P* = 0.032^*^	*P* = 0.121	*P* = 0.022^*^
eGFR _CKD−EPI_ (ml/min/1.73 m^2^)	*r* = 0.169	*r* = 0.140	*r* = 0.142
	*P* = 0.001^*^	*P* = 0.006^*^	*P* = 0.006^*^
CKD groups	*r* = −0.241	*r* = −0.318	*r* = −0.187
	*P* < 0.001^*^	*P* < 0.001^*^	*P* < 0.001^*^
BUN (mmol/L)	*r* = −0.153	*r* = −0.113	*r* = −0.158
	*P* = 0.003^*^	*P* = 0.028^*^	*P* = 0.002^*^
Uric acid (μmol/L)	*r* = −0.071	*r* = −0.078	*r* = −0.020
	*P* = 0.169	*P* = 0.129	*P* = 0.705
Albumin (g/L)	*r* = 0.012	*r* = 0.012	*r* = 0.004
	*P* = 0.824	*P* = 0.811	*P* = 0.934
Protein/creatinine ratio (mg/g Cr)	*r* = −0.093	*r* = −0.065	*r* = −0.113
	*P* = 0.152	*P* = 0.322	*P* = 0.084
Proteinuria (mg/day)	*r* = −0.032	*r* = −0.036	*r* = −0.015
	*P* = 0.605	*P* = 0.567	*P* = 0.810
Triglyceride (mmol/L)	*r* = −0.008	*r* = 0.002	*r* = −0.024
	*P* = 0.874	*P* = 0.965	*P* = 0.648

SCr, serum creatinine; eGFR, estimated glomerular filtration rate; CKD, chronic kidney disease; BUN, blood urea nitrogen; Significant correlation

* *P < 0.05*.

### Association of B1 and B2 Cells With Survival

To assess statistical significance, the X-tile program was employed to determine the cutoff points for B, B1, and B2 cell expression. Using a standard log-rank method, we divided B, B1, and B2 cells into two groups, and the cutoff points for the grouping of B, B1, and B2 cells were 0.05 × 10^9^/L, 0.02 × 10^9^/L, and 0.04 × 10^9^/L, respectively. Then, the OS curves were further analyzed by Kaplan–Meier analysis using SPSS 20.0 software.

**Figure 2:**
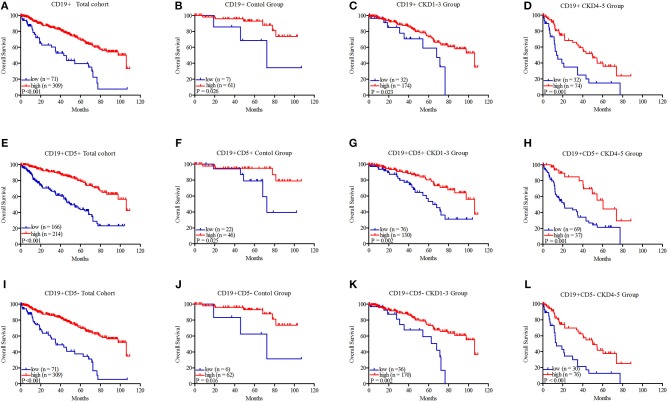
Kaplan–Meier survival curves of CD19+ B lymphocytes, CD19+CD5+ B1 lymphocytes, and CD19+CD5– B2 lymphocytes in the total cohort **(A, E, I)**, control group **(B, F, J)**, CKD1–3 group **(C, G, K)**, and CKD4–5 group **(D, H, L)**. CD19+ B lymphocytes were divided into two groups: low: ≤ 0.05 × 10^9^/L and high: >0.05 × 10^9^/L; CD19+CD5+ B1 lymphocytes were divided into two groups: low: ≤ 0.02 × 10^9^/L and high: >0.02 × 10^9^/L; CD19+CD5– B2 lymphocytes were divided into two groups: low: ≤ 0.04 × 10^9^/L and high: >0.04 × 10^9^/L.

As shown in Figure 2, the patients with B cells > 0.05 × 10^9^/L exhibited better survival in the total cohort (45.62 ± 5.06 vs. 78.95 ± 2.72 months, *P* < 0.001, Figure 2A), the control group (72.08 ± 13.51 vs. 92.69 ± 3.79 months, *P* = 0.026, Figure 2B), the CKD1–3 group (56.70 ± 6.19 vs. 82.64 ± 3.39 months, *P* = 0.023, Figure 2C), and the CKD4–5 group (26.22 ± 5.46 vs. 48.83 ± 4.49 months, *P* = 0.001, Figure 2D). The results of B1 cells were similar; the group with B1 cells > 0.02 × 10^9^/L also showed prolonged survival in the total cohort (53.61 ± 3.65 vs. 86.65 ± 2.96 months, *P* < 0.001, Figure 2E), the control group (76.41 ± 8.15 vs. 97.77 ± 4.24 months, *P* = 0.025, Figure 2F), the CKD1–3 group (64.68 ± 5.34 vs. 86.94 ± 3.68 months, *P* = 0.002, Figure 2G), or the CKD4–5 group (32.55 ± 3.89 vs. 59.39 ± 5.94 months, *P* = 0.001, Figure 2H). The group with B2 cells > 0.04 × 10^9^/L also had better survival in the total cohort (43.54 ± 4.49 vs. 80.00 ± 2.73 months, *P* < 0.001, Figure 2I), the control group (68.78 ± 14.27 vs. 92.76 ± 3.77 months, *P* = 0.016, Figure 2J), the CKD1–3 group (54.79 ± 5.54 vs. 83.87 ± 3.42 months, *P* = 0.002, Figure 2K), or the CKD4–5 group (25.05 ± 4.94 vs. 50.30 ± 4.59 months, *P* < 0.001, Figure 2L).

### Analysis of Prognostic Factors

Univariate analysis showed that CKD3, CKD4, CKD5, B1 cells, B2 cells, NK cells, age, hypertension, systolic pressure, diabetes, hemoglobin, SCr, cystatin C, albumin, and triglyceride were prognostic factors for overall survival. A multivariate Cox regression analysis was then performed for the clinical variables identified as significant in the univariate analysis. We found that CKD3 [hazard ratio (HR) = 2.693, 95% confidence interval (CI): 1.116–6.498, *P* = 0.028], CKD4 (HR = 6.994, 95% CI: 2.708–18.058, *P* < 0.001), CKD5 (HR = 5.772, 95% CI: 2.185–15.244, *P* < 0.001), age (HR = 1.066, 95% CI: 1.022–1.112, *P* = 0.003), and cystatin C (HR = 1.107, 95% CI: 1.033–1.186, *P* = 0.004) were factors significantly associated with a higher risk of all-cause mortality. In contract, B1 cells > 0.02 × 10^9^/L (HR = 0.502, 95% CI: 0.297–0.851, *P* = 0.010) and B2 cells > 0.04 × 10^9^/L (HR = 0.536, 95% CI: 0.319–0.901, *P* = 0.019) were protective factors for survival (Table 3).

**Table 3:** Univariate and multivariate analyses of the prognostic factors.

**Variables**	**Univariate analysis**		**Multivariate analysis**	
	**HR (95% CI)**	***P* value**	**HR (95% CI)**	***P* value**
Control	1	**<0.001**	1	**<0.001**
CKD1-2	1.675 (0.715–3.925)	0.235	2.196 (0.790–6.107)	0.132
CKD3	2.448 (1.176–5.095)	**0.017**	2.693 (1.116–6.498)	**0.028**
CKD4	6.957 (3.184–15.199)	**<0.001**	6.994 (2.708–18.058)	**<0.001**
CKD5	9.334 (4.380–19.894)	**<0.001**	5.772 (2.185–15.244)	**<0.001**
B1 lymphocytes (10^9^/L)				
≤ 0.02	1			
> 0.02	0.292 (0.198–0.430)	**<0.001**	0.502 (0.297–0.851)	**0.010**
B2 lymphocytes (10^9^/L)				
≤ 0.04	1			
> 0.04	0.332 (0.220–0.500)	**<0.001**	0.536 (0.319–0.901)	**0.019**
T lymphocytes (10^9^/L)	0.644 (0.387–1.072)	0.090		
NK cells (10^9^/L)	1.026 (1.005–1.047)	**0.013**		
Female sex	0.755 (0.481–1.185)	0.222		
Age (years)	1.097 (1.059–1.136)	**<0.001**	1.066 (1.022–1.112)	**0.003**
Hypertension	4.223 (1.852–9.629)	**0.001**		
Systolic pressure (mmHg)	1.017 (1.007–1.028)	**0.001**		
Diastolic pressure (mmHg)	1.002 (0.983–1.022)	0.836		
Diabetes	1.490 (1.010–2.198)	**0.044**		
Hemoglobin (g/L)	0.970 (0.962–0.979)	**<0.001**		
Lymphocytes (10^9^/L)	0.710 (0.504–1.001)	0.050		
Monocyte (10^9^/L)	1.212 (0.666–2.205)	0.528		
Triglyceride (mmol/L)	1.137 (1.013–1.276)	**0.030**		
SCr (μmol/L)	1.003 (1.002–1.003)	**<0.001**		
Cystre-C (mg/L)	1.116 (1.072–1.161)	**<0.001**	1.107 (1.033–1.186)	**0.004**
BUN (mmol/L)	1.049 (1.034–1.064)	**<0.001**		
Uric acid (μmol/L)	1.000 (0.998–1.002)	0.799		
Albumin (g/L)	0.947 (0.918–0.976)	**0.001**		
protein/creatinine ratio (mg/g Cr)	1	0.002		
Proteinuria (mg/day)	1	0.001		

*CKD, chronic kidney disease; SCr, serum creatinine; BUN, blood urea nitrogen. Significant correlation: bold values P < 0.05*.

## Discussion

CKD is a major and growing challenge for health care systems. Studies show that CKD is associated with significantly increased morbidity and mortality resulting from cardiovascular disease (CVD) and infections, both of which are linked to immune dysfunction (14). Uremia-related immune dysfunction is a complex interaction between the innate and adaptive systems (15). B lymphocytes are generated from hematopoietic stem cells in bone marrow. IL-7 plays an important role in the B cell developmental pathway by driving pro-B cell maturation in bone marrow (16). Further differentiation of transitional B cells into mature long-lived lymphocytes is critically dependent on the B cell activating factor of tumor necrosis family (BAFF) (17). B cells are classified into different subtypes: B1 cells that secrete IgM and IgA and B2 cells that produce IgG. Previous studies have shown that a low lymphocyte count is a predictor of global death in ESRD patients (10, 18). However, few studies have analyzed the levels of B lymphocyte subpopulations and the relationship between B cell subsets and the clinical features and outcomes of elderly CKD patients.

The geriatric center is the largest geriatric department in South China in our hospital, and we conducted a retrospective study, which made it possible for us to acquire more reliable results. The sex ratio showed that males represented the majority of our patients (76.3%) because most of these patients had been leaders in government or company before, so this research may be more reliable for males. The mean age of our patients was 82.29 years, which was close to a super-elderly age (super-elderly patients were aged more than 85 years). Therefore, our results may provide a reference for the prognostic assessment of elderly patients with CKD.

In this study, we examined a cohort of elderly CKD patients to analyze the distribution and relationship of the B lymphocyte subpopulation with clinical features and all-cause mortality. The main causes of death in our patients were infections (58.7%) and cardiovascular diseases (22.0%). We found that total B, B1, and B2 cells were negatively correlated with SCr levels, BUN levels, and CKD and were positively correlated with lymphocytes, T lymphocytes, and eGFR. Total B cells were also negatively correlated with cystatin C and were positively correlated with NK cells. B2 cells were also negatively associated with age and cystatin C, and were positively correlated with hemoglobin and NK cells. Patient survival was significantly better in those with B cells > 0.05 × 10^9^/L, B1 cells > 0.02 × 10^9^/L, and B2 cells > 0.04 × 10^9^/L. According to the multivariable analysis, age, cystatin C, B1 cells, and B2 cells were associated with all-cause mortality.

In this study, we found a negative correlation between SCr, BUN, CKD, and B and B1 cells, and a positive correlation between B and B1 cells with lymphocyte, T lymphocytes, and eGFR. B cells were also negatively associated with cystatin C and were positively associated with NK cells. Xiang et al. (19) studied CKD patients at different stages and found that B cells were positively correlated with eGFR and were negatively correlated with factors reflecting uremic status, including BUN, cystatin C, and SCr. This is consistent with our results. The relationship between different B cells with CKD and eGFR in our results revealed that B lymphocytes were gradually decreased with the progression of CKD. Previous studies have reported a decreased absolute count of lymphocyte, T lymphocytes, and NK cells along with the deterioration of renal function (8, 9, 20). Accordingly, progressive loss of renal function creates a pro-inflammatory milieu that is highly associated with a dysfunctional immune system. Several studies have demonstrated significant B lymphopenia in patients with ESRD, but these studies were limited to ESRD patients or did not pay attention to elderly patients (7, 8, 10, 21). In our analysis, we studied the distribution and relationship of the B lymphocyte subpopulation with clinical outcomes in CKD stages 1–5 in elderly patients. The decrease in B cells may be associated with B cell apoptosis mediated by resistance to IL-7 and BAFF, both of which are necessary for the differentiation and survival of B cells (7). In addition, the uremic environment may interfere with the maturation of transitional B cells to mature B cells by promoting resistance to BAAF-mediated signals (3). These data indicated that a lower level of B1 cells might be associated with the uremic environment and may be correlated with renal function.

Moreover, our results showed that B2 cells were negatively associated with age, SCr, cystatin C, BUN, and CKD, and were positively associated with hemoglobin, lymphocytes, T lymphocytes, NK cells, and eGFR. B2 production wanes with age (22). Elderly patients typically have lower numbers of B cells due to the reduced output from bone marrow (23). Gibson et al. (24) found that B cell diversity decreased in old age and was correlated with poor health status. Anemia is a complication of renal failure and has been reported to be associated with renal functional prognosis, vascular complications, and death (25). The positive correlation between hemoglobin and B2 cells suggests that immune deficiency may also affect the hematopoietic system. These results revealed that B2 cells were also associated with immune and kidney dysfunction, older age, and a high risk of anemia.

Our study also showed that patients with lower B1 cell and B2 cell counts have a higher risk of all-cause mortality. Several studies have also demonstrated that the incidence of B cell lymphopenia among hemodialysis patients is very high (8, 10). Molina et al. (10) showed that patients with lower CD19+ B cell counts had a higher risk of all-cause and CVD mortality. In our study, we included different stages of elderly CKD patients. We found that CKD-associated immune deficiency was associated with reduced numbers and a reduced antibody producing capacity of B lymphocytes. Immune deficiency leads to impaired responses to vaccination and an increased incidence, severity, and poor outcome of microbial infections. Together, these abnormalities account for the large proportion of morbidity and mortality in patients with advanced CKD (3).

In addition, our data showed that age and cystatin C were also independent risk factors for all-cause mortality in elderly CKD patients. Sun et al. (26) also found that age was a predictor of all-cause mortality in CKD patients. Serum cystatin C is an accurate marker of kidney function and it also has prognostic utility in CKD patients. Bevc et al. (27) found that compared with other kidney function markers, cystatin C had the highest HR for the prediction of outcome in elderly CKD patients. These data are coincident with ours. Although univariate analysis showed that NK cells were a prognostic factor for overall survival, multivariate analysis found that they were not an independent risk factor for prognosis. Therefore, when assessing prognosis and making treatment decisions for the elderly CKD patients, we should consider age and cystatin C.

However, certain limitations of our study should be noted. First, this was a retrospective study. Second, we did not explore the mechanisms of uremia on B cell growth, differentiation, or survival. Further studies should be carried out to explore these results.

## Conclusions

In conclusion, our data demonstrated that B1 and B2 cells were associated with the progression of CKD in elderly patients and B cell lymphopenia could be a predictor of all-cause mortality. These results suggest that B lymphocytes play an important role in immune protection in CKD patients. Further research should focus on the mechanisms of this association and evaluate the possible therapeutic implications.

## Data Availability Statement

All datasets generated for this study are included in the article/supplementary material.

## Ethics Statement

The studies involving human participants were reviewed and approved by the Ethical Committee of Guangdong Provincial People's Hospital. The patients/participants provided their written informed consent to participate in this study.

## Author Contributions

JL was involved in study design, interpreting data, statistical analysis, creating tables and figures, and writing of the manuscript. WT was involved in interpreting data, statistical analysis, and figure and table design. WL, FY, YW, and XF were all involved in data interpretation and critical revisions of the manuscript. MZ took part in flow cytometry analysis. WHa and WHu designed the research, obtained funding, and supervised the work.

### Conflict of Interest

The authors declare that the research was conducted in the absence of any commercial or financial relationships that could be construed as a potential conflict of interest.
